# Adverse Psychological Reactions and Psychological Aids for Medical Staff During the COVID-19 Outbreak in China

**DOI:** 10.3389/fpsyt.2021.580067

**Published:** 2021-04-15

**Authors:** Qinji Su, Xiaoyun Ma, Shun Liu, Shaogang Liu, Bernard A. Goodman, Miaoyu Yu, Wenbin Guo

**Affiliations:** ^1^Mental Health Center, The Second Affiliated Hospital, Guangxi Medical University, Nanning, China; ^2^Department of Epidemiology, School of Public Health, Guangxi Medical University, Nanning, China; ^3^Guangxi Key Laboratory of Chemistry and Engineering of Forest Products, School of Chemistry and Chemical Engineering, Guangxi University for Nationalities, Nanning, China; ^4^School of Physical Science and Technology, Guangxi University, Nanning, China; ^5^Department of Psychiatry, National Clinical Research Center for Mental Disorders, The Second Xiangya Hospital of Central South University, Changsha, China

**Keywords:** COVID-19, medical staff, anxiety, depression, insomnia

## Abstract

**Background:** The outbreak of the novel coronavirus disease COVID-19 caused panic and psychological stress throughout the World. We investigated the extent of adverse psychological reactions in two medical staff groups in China, and explored the importance of online psychological assistance for them.

**Methods:** A cross-sectional online survey including Hospital Anxiety and Depression Scale (HADS) and Insomnia Severity Index (ISI) was utilized to assess anxiety, depression, and insomnia. Propensity score matching (PSM) was applied to match sex and age between the two groups. Differences in the prevalence of adverse psychological reactions between the two groups were compared by a Chi-square test. A multivariate logistic regression analysis was utilized to search for associated adverse psychological reaction factors of two groups.

**Results:** A total of 2,920 medical staff took part in the survey, including 470 frontline and 2,450 non-frontline medical staff. The risk of the frontline group experiencing anxiety, depression, insomnia-early, insomnia-middle, and insomnia-late were 1.16, 1.28, 1.26, 1.22, 1.28 times those of the non-frontline group after PSM. For frontline medical staff, the spinsterhood state (OR = 1.23, 95% CI: 1.00–1.51; *P* = 0.05) was a risk factor for anxiety. Bachelor or college degree (OR = 2.23, 95% CI: 1.24–4.02, *P* = 0.01) and a contact history with COVID-19 patients (OR = 1.62, 95% CI: 1.10–2.40; *P* = 0.02) were risk factors for insomnia. For non-frontline medical staff, being a woman (OR = 1.49, 95% CI: 1.08–2.06, *P* = 0.01) was a risk factor for anxiety, whilst being in a middle age group was a protective factor for anxiety (OR = 0.70, 95% CI: 0.50–0.99, *P* = 0.04) and depression (OR = 0.65, 95% CI: 0.45–0.93, *P* = 0.02). Being a woman (OR = 1.47, 95% CI: 1.14–1.89, *P* = 0.003) and working in a COVID-19 unit (OR = 1.31, 95% CI: 1.11–1.54, *P* = 0.001) were risk factors for insomnia, whilst the spinsterhood state (OR = 0.80, 95% CI: 0.67–0.95; *P* = 0.01) was a protective factor for insomnia. Online forms of psychological aid were all popular with medical staff.

**Conclusions:** The prevalence of anxiety, depression, and insomnia in frontline medical staff was significantly higher than in the non-frontline group. Appropriate intervention methods should be adopted according to the different influencing factors of the two groups. Online psychological aid was the preferred mechanism for relieving psychological problems.

## Introduction

The rapid spread of the novel coronavirus disease COVID-19 resulted in a pandemic affecting more than 100 countries in the first few months of 2020 (1), and created an unprecedented challenge to patients and health care systems (2). According to the World Health Organization (WHO), as of 21 May 2020 confirmed cases numbered 4,893,186 with a death toll of 323,256 (3). In China, a total of 82,971 confirmed cases and 4,634 deaths were reported by the National Health Commission of China for the period to 24:00 on May 20 (4).

The generation of virus-laden respiratory droplets combined with high transmissibility led to rapid human-to-human transmission of COVID-19 (5, 6). Faced with such a critical respiratory infectious disease, varying degrees of anxiety, depression, stress, and psychological reactions were observed in Chinese citizens at the beginning of the outbreak of COVID-19 (7). Furthermore, residents of Hong Kong experienced high perceived susceptibility and severity (8), whilst Twitter users experienced increased anxiety, depression and indignation, and decreased Oxford happiness index (9). In order to alleviate the adverse psychological reactions of social groups, the State Council issued a guideline for a hotline to provide psychological support, counseling, crisis intervention, and other services for various groups involved in epidemic prevention and control (9). However, no specific attention was paid to psychological intervention for medical staff. With the rapid increase in the number of patients with COVID-19 dependent on health care systems, medical staff experienced acute physical and mental burdens, as a result of a soaring workload, separation from families, and fear of becoming infected themselves. This was especially concerning for frontline medical staff who were directly engaged in diagnosis, treatment, and care for patients with COVID-19 (10, 11). Previous studies based on the SARS outbreak in 2003 reported that medical staff suffered adverse psychological reactions, such as, stress, psychological distress, anxiety, depression, and insomnia (12–14), and recent research has suggested that the COVID-19 outbreak posed a huge threat for the development of anxiety, depression, and insomnia in medical staff (15–17). Such negative psychological reactions not only weaken the attention, understanding, and decision-making ability of medical staff, but also result in deterioration in physical health, reluctance to work in potentially dangerous environments, with resignation from hospitals even being considered (18–20).

Anxiety and depression are the most common emotional responses when people are faced with unknown or known threats, which frequently coexist (21, 22). Long periods of anxiety and depression can disrupt normal physiological functions, as well as the immune system (23), and may also be a cause of insomnia (15). Furthermore, poor sleep quality is also detrimental to the functioning of the immune system, and thus, increases vulnerability to the virus (24). On the basis of evidence from the SARS outbreak in 2003, we hypothesized that frontline medical staff might be prone to suffer from anxiety, depression, and insomnia as a result of the high-stress situation of the COVID-19 outbreak, and that it is critical that they receive regular assessments of their mental health status for timely identification of problems and for addressing their psychological status.

Psychological aids from mental health workers can usually detect mental health problems, and provide targeted suggestions for medical staff. However, because of the high transmissibility of COVID-19, there was little free time available for frontline medical staff, and face-to-face counseling was no longer appropriate for them. A recent cross-sectional survey showed that psychotherapy has a major role to relieve the stress level of Spanish healthcare workers during the outbreak of COVID-19 (25). As a consequence, we investigated the contents and forms of psychological aid preferred by medical staff, and our findings may thus provide policy advice for the prevention and treatment of mental health problems in other prolonged high stress situations.

In this study, we aimed to assess the levels of anxiety, depression, and insomnia and compared results between frontline and non-frontline medical staff groups. Moreover, we specifically aimed to identify latent influencing factors of adverse psychological reactions in the two medical staff groups in order to provide evidence for alleviating the severity of anxiety, depression, and insomnia disorders in medical staff in the future. Furthermore, the survey of psychological aid modes should be of value to medical practitioners involved in control of the COVID-19 pandemic.

## Methods

### Study Design and Data Collection

A cross-sectional online survey was designed to assess the mental health status of medical staff. We adopted a free online questionnaire survey platform (SO JUMP; http://www.sojump.com) (26, 27) *via* the WeChat or QQ of the tencent social media network and DingTalk to collect data from respondents. In order to ensure the quality of the questionnaire, we carried out a preliminary survey, and then modified it according to feedback from respondents. The questionnaire information is detailed in Supplementary Materials 1, 2. In order to guarantee confidentiality of personal information, respondents were permitted to answer questionnaires anonymously from 3 to 17 February, 2020. Informed consent was obtained from each participant, and the study was approved by the Ethics Committee of the Second Affiliated Hospital of Guangxi Medical University (No. 2020-KY0004).

### Demographic Information

All subjects enrolled in the survey were medical staff. Demographic information focused on sex (men and women), age (≤28, 29–40, >40), educational level (<Undergraduate/junior college, Undergraduate/junior college, ≥Postgraduate), marital status (widowed/divorced, married, single), medical staff group (frontline and non-frontline), region (non-risk, low-risk, medium-risk, and high-risk), history of contact with patients with COVID-19 (positive and negative), and the COVID-19 unit (positive and negative). The frontline medical staff were engaged directly in diagnosis, treatment, and care for patients with COVID-19. The classification of zone was based on the epidemic risk level query website (https://bmfw.www.gov.cn/yqfxdjcx/index.html). Most subjects enrolled in the present study were from a low-risk zone. Positive COVID-19 unit meant this hospital received and treated patients with COVID-19.

### Hospital Anxiety and Depression Scale

The Chinese version of HADS was used to identify the presence of anxiety and depression disorder in the medical staff (28). This questionnaire comprised two subscales of anxiety (HADS-A) and depression (HADS-D), and each subscale contained seven self-assessment screening items. The score of each item was deemed as 0 (never), 1 (mild), 2 (moderate), or 3 (severe). Overall, the total scores of HADS-A and HADS-D were classified as normal (0–7) and anxiety or depressive (8–21) with higher scores indicating higher levels of symptoms.

### Insomnia

In order to investigate whether the respondents had symptoms of insomnia and to assess its severity, we designed four questions according to the Chinese version of the Insomnia Severity Index (ISI) (29, 30); these were classified as insomnia-early, insomnia-middle, and insomnia-late. Insomnia - early means difficulty initiating sleep, insomnia-middle means difficulty maintaining sleep, and insomnia-late means waking up too early and not being able to fall back asleep. In addition to these questions, we attempted to evaluate sleep quality through questions of sleep mode satisfaction. The answer to each question was evaluated as 0 (never), 1 (mild), 2 (moderate), 3 (severe), or 4 (extremely severe), and classified in two levels: normal (0) and abnormal (1–4).

### Psychological Aid

We designed three questions to probe whether psychological aid was necessary in order to perform medical work, and assessed the need for psychological aid, along with its forms and contents. For the questions on “the forms of psychological aid” and “the contents of psychological aid,” participants were able to choose more than one option.

### Statistical Analysis

Respondents were divided into frontline and non-frontline medical staff groups, and their demographic information and mental health status scores were presented as frequency distributions (numbers and percentages). The 1:1 ratio propensity score matching (PSM) method was applied to match sociodemographic characteristics such as sex and age between frontline and non-frontline medical staff groups in order to eliminate the influence of confounding factors. The statistical magnitude of the L1 measure was used to evaluate the effect of matching. The statistical magnitude of L1 measure was lower, and the effect of matching was improved.

A Chi-square test was used to determine if there were significant differences in prevalence for anxiety, depression, and insomnia symptoms between the frontline and non-frontline medical staff groups. A Spearman's rank correlation was conducted to explore any relationship between the anxiety and depression symptoms. Furthermore, we used stratified analyses to explore the correlative and influencing factors of adverse psychological reactions in two groups. First of all, the potential associated factors for adverse psychological reactions of the two groups was performed by Chi-square test. Then, we conducted a multivariate logistic regression analysis to seek out potential influencing factors for anxiety, depression, and insomnia symptoms in two groups. The odds ratios (ORs) and 95% confidence interval (CI) were applied to describe the relationship between mental health status and influencing factors. SPSS 22.0 software (IBM, Armonk, NY, USA) was applied to analysis all of the statistical results, which were plotted using GraphPad Prism 8.0 (La Jolla, CA, USA). All analyses were two sided, with *P* < 0.05 considered to be statistically significant.

## Results

### Demographic Characteristics

In general, a total of 2,920 eligible questionnaires were collected from 470 (16.1%) frontline and 2,450 (83.9%) non-frontline medical staff. The majority of participants were women (86.8%). We divided them into three age groups. The middle age group (29–40) had the largest proportion (51.4%), followed by the younger age group (33.1%) and the older age group (15.5%). The majority of their academic qualifications were undergraduate or junior college (85.4%). Married persons accounted for the largest proportion (66.4%), and 2,337 (80.0%) respondents lived in non-risk regions, whilst 583 (20.0%) respondents admitted that they had been exposed to confirmed or suspected cases of COVID-19. Furthermore, 1,766 (60.5%) work units of participants administered and treated patients with COVID-19. Full demographic details are shown in Table 1.

**Table 1:** Comparison of basic information between frontline and non-frontline medical staff.

**Basic information**	**Total (%)**	**The medical staff group**		**χ2**	***P***
		**Frontline**	**Non-frontline**		
Overall(%)	2,920 (100%)	470 (16.1%)	2,450 (83.9%)		
Sex				49.00	**<0.001**
Men	385 (13.2%)	109 (23.2%)	276 (11.3%)		
Women	2,535 (86.8%)	361 (76.8%)	2,174 (88.7%)		
Age				10.13	**0.01**
≤28	967 (33.1%)	132 (28.1%)	835 (34.1%)		
29–40	1,501 (51.4%)	273 (58.1%)	1,228 (50.1%)		
>40	452 (15.5%)	65 (13.8%)	387 (15.8%)		
Education level				2.96	0.23
<Undergraduate/junior college	150 (5.1%)	18 (3.9%)	132 (5.4%)		
Undergraduate/junior college	2,493 (85.4%)	401 (85.3%)	2,092 (85.4%)		
≥Postgraduate	277 (9.5%)	51 (10.9%)	226 (9.2%)		
Marital status				0.88	0.65
Widowed/divorced	74 (2.5%)	11 (2.3%)	63 (2.6%)		
Married	1,940 (66.4%)	321 (68.3%)	1,619 (66.1%)		
Spinsterhood	906 (31.0%)	138 (29.4%)	768 (31.3%)		
Region				35.98	**<0.001**
No-risk region	2,337 (80.0%)	346 (73.6%)	1,991 (81.3%)		
Low-risk region	31 (1.1%)	9 (1.9%)	22 (0.9%)		
Medium-risk region	538 (18.4%)	106 (22.6%)	432 (17.6%)		
High-risk region	14 (0.5%)	9 (1.9%)	5 (0.2%)		
Contact history				443.43	**<0.001**
Positive	583 (20.0%)	261 (55.5%)	322 (13.1%)		
Negative	2,337 (80.0%)	209 (44.5%)	2,128 (86.9%)		
COVID-19 work unit				59.28	**<0.001**
Positive	1,766 (60.5%)	359 (76.4%)	1,407 (57.4%)		
Negative	1,154 (39.5%)	111 (23.6%)	1,043 (42.6%)		

*The bold values indicate that the differences are statistically significant*.

We divided the participants into frontline and non-frontline medical staff groups, the ratio of men to women is about 1:3 in frontline medical staff, while the ratio is about 1:8 in non-frontline medical staff group. There is a statistical difference in sex between two groups by the Chi-square test (χ^2^ = 49.00, *df* = 1, *P* < 0.001). Similarly, the distribution of age was different between the two groups, and the difference was also statistically significant (χ^2^ = 10.13, *df* = 2, *P* = 0.006). However, the distribution of the education level and marital status were not statistically significant between two groups (χ^2^ = 2.96, *df* = 2, *P* = 0.23; χ^2^ = 0.88, *df* = 2, *P* = 0.65). Therefore, we matched the two groups by sex and age through propensity score matching (PSM). The results showed that 470 frontline medical staff and 470 non-frontline medical staff were matched through sex and age. The statistical magnitude of L1 measure was reduced after matching (0.14 vs. 0.01), and the difference in sex and age was not statistically significant (both χ^2^ < 0.001, *df* = 1 or *df* = 2, *P* = 1.00) after matching, indicating it was a good PSM.

### Comparisons of the Symptoms of Adverse Psychological Reactions Between Frontline and Non-frontline Groups After PSM

As shown in Table 2, the proportion of frontline medical staff experiencing anxiety was higher than for non-frontline medical staff (30.0 vs. 24.3%). Result unveiled that frontline medical staff may be more prone to anxiety compared to the non-frontline medical staff (χ^2^ = 3.92, *df* = 1, *P* = 0.05). As for depression, 123 (26.2%) frontline medical staff suffered varying degrees of depression, whereas, 86 (18.3%) non-frontline medical staff admitted to having similar symptoms. Statistical results indicate that the frontline medical staff may be more prone to depression (χ^2^ = 8.42, *df* = 1, *P* = 0.004). Furthermore, Spearman's rank correlation showed a positive correlation between the total scores for anxiety and depression (*r*_*s*_ = 0.75, *df* = 2918, *P* < 0.001), suggesting that medical staff may suffer from depression accompanying anxiety.

**Table 2:** Comparisons of the symptoms of adverse psychological reactions between frontline and non-frontline groups after PSM.

**Adverse psychological reactions**	**Total (%)**	**The medical staff group**		**OR**	**OR95% CI**	**χ2**	***P***
		**Frontline**	**Non-frontline**				
*N*	940	470	470				
Anxiety						3.92	0.05
Normal	685 (72.9%)	329 (70.0%)	356 (75.7%)	1.00 (reference)		
Abnormal	255 (27.1%)	141 (30.0%)	114 (24.3%)	1.16	1.00–1.36		
Depression						8.42	0.004
Normal	731 (77.8%)	347 (73.8%)	384 (81.7%)	1 (reference)		
Abnormal	209 (22.2%)	123 (26.2%)	86 (18.3%)	1.28	1.07–1.52		
Insomnia-early						12.53	<0.001
Normal	424 (45.1%)	185 (39.4%)	239 (50.9%)	1 (reference)		
Abnormal	516 (54.9%)	285 (60.6%)	231 (49.1%)	1.26	1.11–1.43		
Insomnia-middle						9.42	0.002
Normal	489 (52.0%)	221 (47.0%)	268 (57.0%)	1 (reference)		
Abnormal	451 (48.0%)	249 (53.0%)	202 (43.0%)	1.22	1.07–1.39		
Insomnia-late						13.83	<0.001
Normal	473 (50.3%)	208 (44.3%)	265 (56.4%)	1 (reference)		
Abnormal	467 (49.7%)	262 (55.7%)	205 (43.6%)	1.28	1.12–1.45		
Sleep mode satisfaction						5.14	0.02
Normal	158 (16.8%)	66 (14.0%)	92 (19.6%)	1 (reference)		
Abnormal	782 (83.2%)	404 (86.0%)	378 (80.4%)	1.21	1.04–1.40		

*PSM, Propensity Score Matching*.

In addition, 60.6% (285) of frontline medical staff suffered from varying degrees of insomnia-early, whereas, the corresponding proportion of non-frontline medical staff was 49.1% (231), the difference was statistically significant (χ^2^ = 12.53, *df* = 1, *P* < 0.001). 53.0% (249) of frontline medical staff suffered from varying degrees of insomnia-middle, compared to 43.0% (202) of non-frontline medical staff, the difference was statistically significant (χ^2^ = 9.42, *df* = 1, *P* = 0.002). Similarly, the proportion of frontline medical staff was higher than non-frontline medical staff for insomnia late (55.7 vs. 43.6%). The difference was statistically significant (χ^2^ = 13.83, *df* = 1, *P* < 0.001). For sleep mode satisfaction, more frontline than non-frontline medical staff expressed dissatisfaction with sleep patterns (86.0 vs. 80.4%), and this result was highly significant (χ^2^ = 5.14, *df* = 1, *P* = 0.02). Thus, overall frontline medical staff had more problems with sleeping.

### Potential Correlative Factors for Anxiety and Depression in Two Medical Staff Groups by Stratification Analysis

We used stratified analyses to explore the correlative factors of anxiety and depression in two groups. The Chi-square test analysis showed that only the marital status was related with the occurrence of anxiety (χ^2^ = 7.13, *df* = 2, *P* = 0.03), while other factors were not associated with the symptom of anxiety among frontline medical staff. For the symptom of depression, we failed to identify the factors associated with depression among frontline group.

For non-frontline medical staff, the sex (χ^2^ = 5.20, *df* = 1, *P* = 0.02), the age (χ^2^ = 9.05, *df* = 2, *P* = 0.01), and the marital status (χ^2^ = 5.83, *df* = 2, *P* = 0.05) were related to the incidence of anxiety. Also, the age (χ^2^ = 13.17, *df* = 2, *P* = 0.001), the education level (χ^2^ = 6.41, *df* = 2, *P* = 0.04), and the marital status (χ^2^ = 7.30, *df* = 2, *P* = 0.03) were related to the incidence of depression. The detail information are shown in Table 3.

**Table 3:** Factors associated with the symptoms of anxiety and depression in two groups.

	**Frontline medical staff**								**Non-frontline medical staff**							
	**Anxiety**		**χ2**	***P***	**Depression**		**χ2**	***P***	**Anxiety**		**χ2**	***P***	**Depression**		**χ2**	***P***
	**Normal**	**Abnormal**			**Normal**	**Abnormal**			**Normal**	**Abnormal**			**Normal**	**Abnormal**		
*N*	329	141			347	123			1,862	588			1,968	482		
Sex			0.42	0.52			0.14	0.70			5.20	**0.02**			1.38	0.24
Men	79 (24.0%)	30 (21.3%)			82 (23.6%)	27 (22.0%)			225 (12.1%)	51 (8.7%)			229 (11.6%)	47 (9.8%)		
Women	250 (76.0)	111 (78.7)			265 (76.4%)	96 (78.0%)			1,637 (87.9%)	537 (91.3%)			1,739 (88.4%)	435 (90.2%)		
Age			3.42	0.18			0.25	0.88			9.05	**0.01**			13.17	**0.001**
≤28	102 (31.0%)	32 (22.7%)			101 (29.1%)	33 (26.8%)			666 (35.8%)	171 (29.1%)			703 (35.7%)	134 (27.8%)		
29–40	184 (55.9%)	87 (61.7)			198 (57.1%)	73 (59.3%)			912 (49.0%)	314 (53.4%)			972 (49.4%)	254 (52.7%)		
>40	43 (13.1%)	22 (15.6)			48 (13.8%)	17 (13.8%)			284 (15.3%)	103 (17.5%)			293 (14.9%)	94 (19.5%)		
Education level			1.84	0.40			3.00	0.22			0.32	0.85			6.41	**0.04**
<Undergraduate/junior college	15 (4.6%)	3 (2.1%)			16 (4.6%)	2 (1.6%)			103 (5.5%)	29 (4.9%)			100 (5.1%)	32 (6.6%)		
Undergraduate/junior college	277 (84.2%)	124 (87.9%)			291 (83.9%)	110 (89.4%)			1,588 (85.3%)	504 (85.7%)			1,698 (86.3%)	394 (81.7%)		
≥Postgraduate	37 (11.2%)	14 (9.9%)			40 (11.5%)	11 (8.9%)			171 (9.2%)	55 (9.4%)			170 (8.6%)	56 (11.6%)		
Marital status			7.13	**0.03**			2.69	0.26			5.83	**0.05**			7.30	**0.03**
Widowed/divorced	6 (1.8%)	5 (3.5%)			8 (2.3%)	3 (2.4%)			43 (2.3%)	20 (3.4%)			45 (2.3%)	18 (3.7%)		
Married	215 (65.3%)	106 (75.2%)			230 (66.3%)	91 (74.0%)			1,215 (65.3%)	404 (68.7%)			1,286 (65.3%)	333 (69.1%)		
Spinsterhood	108 (32.8%)	30 (21.3%)			109 (31.4%)	29 (23.6%)			604 (32.4%)	164 (27.9%)			637 (32.4%)	131 (27.2%)		
Region			1.11	0.77			1.81	0.61			3.24	0.36			4.02	0.26
No-risk region	238 (72.3%)	108 (76.6%)			260 (74.9%)	86 (69.9%)			1,527 (82.0%)	464 (78.9%)			1,614 (82.0%)	377 (78.2%)		
Low-risk region	7 (2.1%)	2 (1.4%)			7 (2.0%)	2 (1.6%)			15 (0.8%)	7 (1.2%)			18 (0.9%)	4 (0.8%)		
Medium-risk region	77 (23.4%)	29 (20.6%)			73 (21.0%)	33 (26.8%)			316 (17.0%)	116 (19.7%)			332 (16.9%)	100 (20.7%)		
High-risk region	7 (2.1%)	2 (1.4%)			7 (2.0%)	2 (1.6%)			4 (0.2%)	1 (0.2%)			4 (0.2%)	1 (0.2%)		
Contact history			0.02	0.89			2.00	0.16			2.69	0.10			2.11	0.15
Positive	182 (55.3%)	79 (56.0%)			186 (53.6%)	75 (61.0%)			233 (12.5%)	89 (15.1%)			249 (12.7%)	73 (15.1%)		
Negative	147 (44.7%)	62 (44.0%)			161 (46.4%)	48 (39.0%)			1,629 (87.5%)	499 (84.9%)			1,719 (87.3%)	409 (84.9%)		
COVID-19 work unit			0.01	0.94			0.00	0.99			2.15	0.14			1.10	0.30
Positive	251 (76.3%)	108 (76.3%)			265 (76.4%)	94 (76.4)			1,054 (56.6%)	353 (60.0%)			1,120 (56.9%)	287 (59.5%)		
Negative	78 (23.7%)	33 (23.7%)			82 (23.6%)	29 (23.6%)			808 (43.4%)	235 (40.0%)			848 (43.1%)	195 (40.5%)		

*The bold values indicate that the differences are statistically significant*.

### Potential Correlative Factors for Insomnia in Two Medical Staff Groups by Stratification Analysis

Among frontline medical staff, the education level was connected with insomnia-early (χ^2^ = 7.36, *df* = 2, *P* = 0.03). Whereas, the education level (χ^2^ = 5.86, *df* = 2, *P* = 0.05), the contact history (χ^2^ = 9.68, *df* = 1, *P* = 0.002) and working in COVID-19 work unit (χ^2^ = 6.60, *df* = 1, *P* = 0.01) were related to insomnia-middle. However, we failed to find out the factors associated with insomnia-late and sleep mode satisfaction in frontline medical staff. The detail information were shown in Table 4.

**Table 4:** Factors associated with the symptoms of insomnia in two groups.

	**Frontline medical staff**																**Non-frontline medical staff**															
	**Insomnia-early**		**χ2**	***P***	**Insomnia-middle**		**χ2**	***P***	**Insomnia-late**		**χ2**	***P***	**Sleep mode satisfaction**		**χ2**	***P***	**Insomnia-early**		**χ2**	***P***	**Insomnia-middle**		**χ2**	***P***	**Insomnia-late**		**χ2**	***P***	**Sleep mode satisfaction**		**χ2**	***P***
	**Normal**	**Abnormal**			**Normal**	**Abnormal**			**Normal**	**Abnormal**			**Normal**	**Abnormal**			**Normal**	**Abnormal**			**Normal**	**Abnormal**			**Normal**	**Abnormal**			**Normal**	**Abnormal**		
*N*	185	285			221	249			208	262			66	404			1,166	1,284			1,365	1,085			1,321	1,129			454	1,996		
Sex			0.48	0.49			0.03	0.87			0.003	0.96			0.05	0.83			10.77	**0.001**			8.93	**0.003**			2.86	0.09			10.32	**<0.001**
Men	46 (24.9%)	63 (22.1%)			52 (23.5%)	57 (22.9%)			48 (23.1%)	61 (23.3%)			16 (24.2%)	93 (23.0%)			157 (13.5%)	119 (9.3%)			177 (13.0%)	99 (9.1%)			162 (12.3%)	114 (10.1%)			75 (16.5%)	201 (10.1%)		
Women	139 (75.1%)	222 (77.9%)			169 (76.5%)	192 (77.1%)			160 (76.9%)	201 (76.7%)			50 (75.8%)	311 (77.0%)			1,009 (86.5%)	1,165 (90.7%)			1,188 (87.0%)	986 (90.9%)			1,159 (87.7%)	1,015 (89.9%)			379 (83.5%)	1,795 (89.9%)		
Age			3.30	0.19			2.79	0.25			0.31	0.86			0.52	0.77			1.17	0.56			4.08	0.13			30.79	**<0.001**			0.70	0.71
≤28	45 (24.3%)	89 (31.2%)			56 (25.3%)	78 (31.3%)			62 (29.8%)	72 (27.5%)			18 (27.3%)	116 (28.7)			386 (33.1%)	451 (35.1%)			484 (35.5%)	353 (32.5%)			489 (37.0%)	348 (30.8%)			151 (33.3%)	686 (34.4%)		
29–40	110 (59.5%)	161 (56.5%)			130 (58.8%)	141 (56.6%)			118 (56.7%)	153 (58.4%)			37 (56.1%)	234 (57.9%)			595 (51.0%)	631 (49.1%)			681 (49.9%)	545 (50.2%)			671 (50.8%)	555 (49.2%)			235 (51.8%)	991 (49.6%)		
>40	30 (16.2%)	35 (12.3%)			35 (15.8%)	30 (12.0%)			28 (13.5%)	37 (14.1%)			11 (16.7%)	54 (13.4%)			185 (15.9%)	202 (15.7%)			200 (14.7%)	187 (17.2%)			161 (12.2%)	226 (20.0%)			68 (15.0%)	319 (16.0%)		
Education level			7.36	**0.03**			5.86	**0.05**			4.36	0.11			3.75	0.15			3.33	0.19			3.04	0.22			2.95	0.23			7.14	**0.03**
<Undergraduate/junior college	7 (3.8%)	11 (3.9%)			9 (4.1%)	9 (3.6%)			6 (2.9%)	12 (4.6%)			5 (7.6%)	13 (3.2%)			59 (5.1%)	73 (5.7%)			75 (5.5%)	57 (5.3%)			63 (4.8%)	69 (6.1%)			20 (4.4%)	112 (5.6%)		
Undergraduate/junior college	149 (80.5%)	252 (88.4%)			180 (81.4%)	221 (88.8%)			173 (83.2%)	228 (87.0%)			52 (78.8%)	349 (86.4%)			987 (84.6%)	1,105 (86.1%)			1,152 (84.4%)	940 (86.6%)			1,129 (85.5%)	963 (85.3%)			378 (83.3%)	1,714 (85.9%)		
≥Postgraduate	29 (15.7%)	22 (7.7%)			32 (14.5%)	19 (7.6%)			29 (13.9%)	22 (8.4%)			9 (13.6%)	42 (10.4%)			120 (10.3%)	106 (8.3%)			138 (10.1%)	88 (8.1%)			129 (9.8%)	97 (8.6%)			56 (12.3%)	170 (8.5%)		
Marital status			2.10	0.35			1.71	0.43			0.05	0.98			0.78	0.68			8.13	**0.02**			0.70	0.71			8.37	**0.02**			0.59	0.74
Widowed/divorced	6 (3.2%)	5 (1.8%)			7 (3.2%)	4 (1.6%)			5 (2.4%)	6 (2.3%)			1 (1.5%)	10 (2.5%)			24 (2.1%)	39 (3.0%)			32 (2.3%)	31 (2.9%)			25 (1.9%)	38 (3.4%)			11 (2.4%)	52 (2.6%)		
Married	130 (70.3%)	191 (67.0%)			153 (69.2%)	168 (67.5%)			141 (67.8%)	180 (68.7%)			48 (72.7%)	273 (67.6%)			802 (68.8%)	817 (63.6%)			907 (66.4%)	712 (65.6%)			859 (65.0%)	760 (67.3%)			307 (67.6%)	1,312 (65.7%)		
Spinsterhood	49 (26.5%)	89 (31.2%)			61 (27.6%)	77 (30.9%)			62 (29.8%)	76 (29.0%)			17 (25.8%)	121 (30.0%)			340 (29.2%)	428 (33.3%)			426 (31.2%)	342 (31.5%)			437 (33.1%)	331 (29.3%)			136 (30.0%)	632 (31.7%)		
Region			3.07	0.38			1.45	0.69			4.69	0.20			3.68	0.30			1.16	0.76			0.52	0.92			4.17	0.24			1.97	0.58
No-risk region	136 (73.5%)	210 (73.7%)			162 (73.3%)	184 (73.9%)			150 (72.1%)	196 (74.8%)			45 (68.2%)	301 (74.5%)			954(81.8%)	1,037 (80.8%)			1,110 (81.3%)	881 (81.2%)			1,059 (80.2%)	932 (82.6%)			374 (82.4%)	1,617 (81.0%)		
Low-risk region	6 (3.2%)	3 (1.1%)			6 (2.7%)	3 (2.1%)			7 (3.4%)	2 (0.8%)			3 (4.5%)	6 (1.5%)			12 (1.0%)	10 (0.8%)			12 (0.9%)	10 (0.9%)			10 (0.8%)	12 (1.1%)			6 (1.3%)	16 (0.8%)		
Medium-risk region	40 (21.6%)	66 (23.2%)			49 (22.2%)	57 (22.9%)			48 (23.1%)	58 (22.1%)			16 (24.2%)	90 (22.3%)			198 (17.0%)	234 (18.2%)			241 (17.7%)	191 (17.6%)			250 (18.9%)	182 (16.1%)			73 (16.1%)	359 (18.0%)		
High-risk region	3 (1.6%)	6 (2.1%)			4 (1.8%)	5 (2.0%)			3 (1.4%)	6 (2.3%)			2 (3.0%)	7 (1.7%)			2 (0.2%)	3 (0.2%)			2 (0.1%)	3 (0.3%)			2 (0.2%)	3 (0.3%)			1 (0.2%)	4 (0.2%)		
Contact history			3.42	0.06			9.68	**0.002**			0.08	0.78			0.50	0.48			0.001	0.98			1.88	0.17			8.72	**0.003**			1.05	0.31
Positive	93 (50.3%)	168 (58.9%)			106 (48.0%)	155 (62.2%)			117 (56.3%)	144 (55.0%)			34 (51.5%)	227 (56.2%)			153 (13.1%)	169 (13.2%)			168 (12.3%)	154 (14.2%)			149 (11.3%)	173 (15.3%)			53 (11.7%)	269 (13.5%)		
Negative	92 (49.7%)	117 (41.1%)			115 (52.0%)	94 (37.8%)			91 (43.8%)	118 (45.0)			32 (48.5%)	177 (43.8%)			1,013 (86.9%)	1,115 (86.8%)			1,197 (87.7%)	931 (85.8)%			1,172 (88.7%)	956 (84.7%)			401 (88.3%)	1,727 (86.5%)		
COVID-19 work unit			1.97	0.16			6.60	**0.01**			2.97	0.09			0.20	0.66			11.59	**0.001**			14.26	**<0.001**			13.99	**<0.001**			9.13	**0.003**
Positive	135 (73.0%)	224 (78.6%)			157 (71.0%)	202 (81.1%)			151 (72.6%)	208 (79.4%)			49 (74.2%)	310 (76.7%)			628 (53.9%)	779 (60.7%)			738 (54.1%)	669 (61.7%)			713 (54.0%)	694 (61.5%)			232 (51.1%)	1,175 (58.9%)		
Negative	50 (27.0%)	61 (21.4%)			64 (29.0%)	47 (18.9%)			57 (27.4%)	54 (20.6%)			17 (25.8%)	94 (23.3%)			538 (46.1%)	505 (39.3%)			627 (45.9%)	416 (38.3%)			608 (46.0%)	435 (38.5%)			222 (48.9%)	821 (41.1%)		

*The bold values indicate that the differences are statistically significant*.

Among non-frontline medical staff, the sex (χ^2^ = 10.77, *df* = 1, *P* = 0.001), the marital status (χ^2^ = 8.13, *df* = 2, *P* = 0.02), and working in COVID-19 work unit (χ^2^ = 11.59, *df* = 1, *P* = 0.001) were associated with insomnia-early. Whereas, the sex (χ^2^ = 8.93, *df* = 1, *P* = 0.003) and working in COVID-19 work unit (χ^2^ = 14.26, *df* = 1, *P* < 0.001) were correlated to insomnia-middle. Also, we found that the age (χ^2^ = 30.79, *df* = 2, *P* < 0.001), the marital status (χ^2^ = 8.37, *df* = 2, *P* = 0.02), the contact history (χ^2^ = 8.72, *df* = 1, *P* = 0.003), and working in COVID-19 work unit (χ^2^ = 13.99, *df* = 1, *P* < 0.001) were related to insomnia-late. What's more, we discovered that the sex (χ^2^ = 10.32, *df* = 1, *P* < 0.001), the education level (χ^2^ = 7.14, *df* = 2, *P* = 0.03), and working in COVID-19 unit (χ^2^ = 9.13, *df* = 1, *P* = 0.003) were correlated to sleep mode satisfaction. The detail information are shown in Table 4.

### Potential Influencing Factors of Adverse Psychological Reactions of Two Medical Staff by Stratification Analysis

Among frontline and non-frontline medical staff, we found a series of factors that were related to anxiety, depression, and insomnia by univariate analysis. Therefore, we used the statistically significant variables obtained from univariate analysis to conduct a further multivariate logistic regression to find out the latent influencing factors of two groups. For frontline medical staff, the results unveiled that the spinsterhood state (OR = 1.23, 95% CI: 1.00–1.51; *P* = 0.05) was a risk factor for anxiety compared to widowed/divorced. Bachelor or college degree was the risk factor for insomnia-early (OR = 2.23, 95% CI: 1.24–4.02, *P* = 0.01) and insomnia-middle (OR = 2.01, 95% CI: 1.09–3.69; *P* = 0.03). In addition, the COVID-19 patients contact history (OR = 1.62, 95% CI: 1.10–2.40; *P* = 0.02) was a risk factor for insomnia-middle. The detail information are shown in Table 5.

**Table 5:** Factors of influencing adverse psychological reactions in frontline group by logistic regression analysis.

	**β**	**SE**	**Wald**	**OR**	**OR 95% CI**	***P***
**Anxiety**						
Marital status						
Widowed/divorced (control)						
Married	0.54	0.29	3.58	1.71	0.98–2.99	0.06
Spinsterhood	0.20	0.11	3.72	1.23	1.00–1.51	**0.05**
**Insomnia-early**						
Education level						
<Undergraduate/junior college						
(control)						
Undergraduate/junior college	0.73	0.56	1.69	2.07	0.69–6.21	0.19
≥Postgraduate	0.80	0.30	7.09	2.23	1.24–4.02	**0.01**
**Insomnia-middle**						
Education level						
<Undergraduate/junior college						
(control)						
Undergraduate/junior college	0.72	0.56	1.61	2.04	0.68–6.17	0.21
≥Postgraduate	0.70	0.31	5.05	2.01	1.09–3.69	**0.03**
Contact history						
Negative (control)						
Positive	0.48	0.20	5.89	1.62	1.10–2.40	**0.02**
COVID-19 work unit						
Negative (control)						
Positive	0.37	0.23	2.50	1.45	0.92–2.29	0.11

*The bold values indicate that the differences are statistically significant*.

For non-frontline medical staff, the women (OR = 1.49, 95% CI: 1.08–2.06, *P* = 0.01) was a risk factor for the occurrence of anxiety, while middle age group was a protective factor not only for anxiety (OR = 0.70, 95% CI: 0.50–0.99, *P* = 0.04) but also for depression (OR = 0.65, 95% CI: 0.45–0.93, *P* = 0.02). The women (OR = 1.47, 95% CI: 1.14–1.89, *P* = 0.003) and working in a COVID-19 unit (OR = 1.31, 95% CI: 1.11–1.54, *P* = 0.001) were risk factors for insomnia-early, while the spinsterhood state (OR = 0.80, 95% CI: 0.67–0.95; *P* = 0.01) was a protective factor for insomnia-early. Also, the women (OR = 1.47, 95% CI: 1.14–1.90, *P* = 0.003) and working in a COVID-19 unit (OR = 1.30, 95% CI: 1.10–1.52, *P* = 0.002) were risk factors for insomnia-middle. Working in a COVID-19 unit (OR = 1.37, 95% CI:1.16–1.61 *P* < 0.001) was a risk factor for insomnia-late, while the spinsterhood state (OR = 0.76, 95% CI: 0.61–0.95; *P* = 0.02) was a protective factor for insomnia-late. As for sleep mode satisfaction, the women (OR = 1.58, 95% CI: 1.17–2.15; *P* = 0.003) and working in a COVID-19 unit (OR = 1.33, 95% CI: 1.09–1.64, *P* = 0.01) were risk factors. The detail information are shown in Supplementary Table 1.

### Psychological Aid

**Figure 1:**
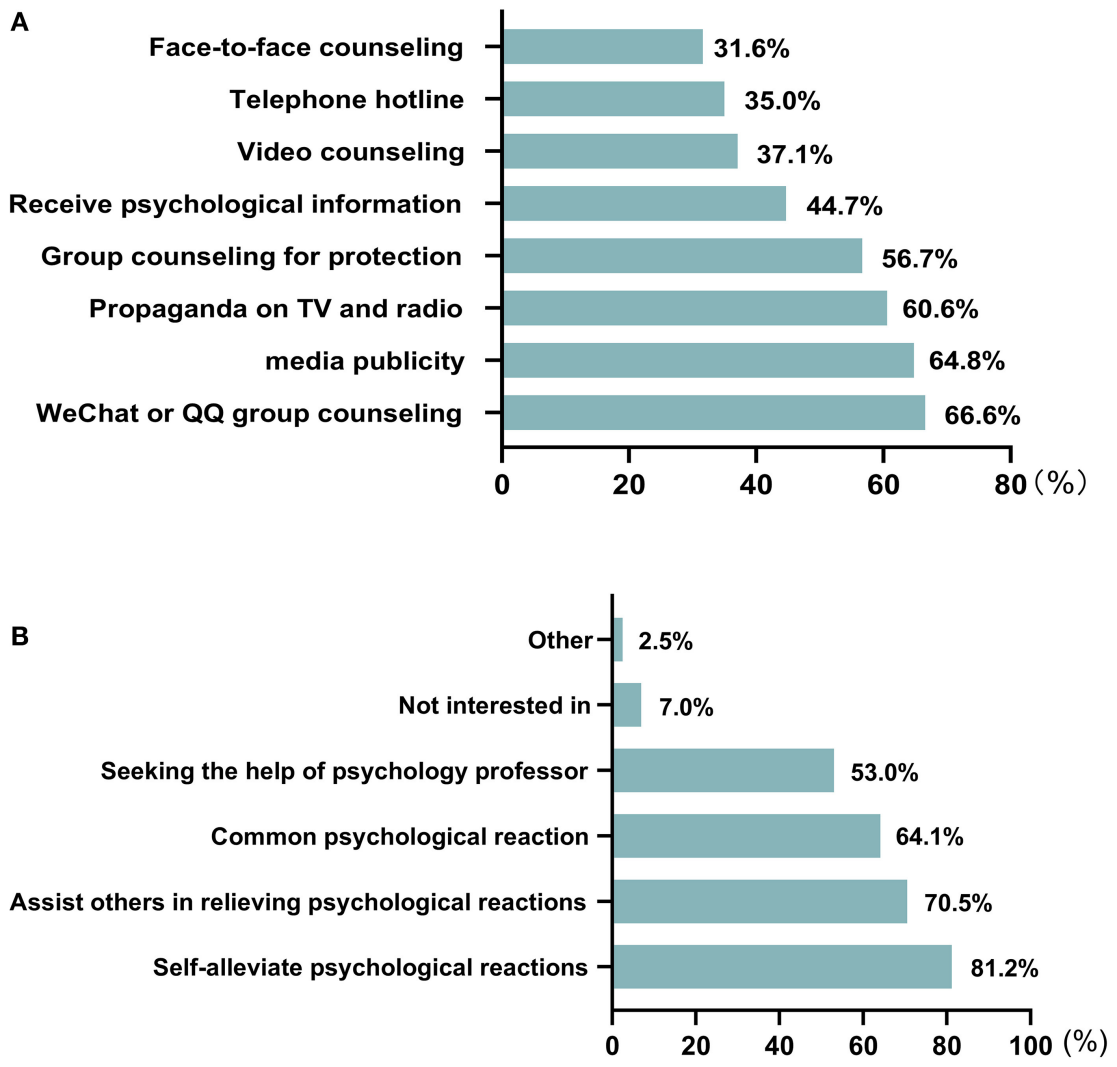
Findings on the psychological aid needs. **(A)** The choice of medical staff for psychological aid forms. **(B)** The choice of medical staff for content of psychological aid.

As a result of the high-pressure working environment during the COVID-19 pandemic, medical staff were susceptible to psychological problems. Therefore, we further investigated the need for psychological aid for all medical staff. The results showed that 53.0% (1,526) thought it necessary for medical staff to provide and receive psychological help. The provision of online forms for psychological aid, WeChat or QQ group counseling (66.6%), public account publicity (64.8%), and propaganda on TV and radio (60.6%) were the three most popular procedures (Figure 1A). Furthermore, medical staff were more inclined to be familiar with “how to self-alleviate psychological reactions” (81.2%), “how to help others relieve psychological reactions” (70.5%) and “common psychological reactions” (64.1%) (Figure 1B).

## Discussion

In the present study, we observed that a number of medical staff suffered from adverse psychological reactions to varying degrees during the outbreak of COVID-19. The results showed that frontline medical staff were at greater risk for anxiety, depression, and insomnia compared to non-frontline medical staff, and that there was an increased prevalence of anxiety and depression disorders at the height of the COVID-19 pandemic. Similar conclusions that frontline medical staff in high-risk departments were more susceptible to feelings of anxiety, depression, and insomnia compared with non-frontline staff have also been reported (16, 31–34). However, we also discovered a positive correlation between anxiety and depression, and the co-existence of anxiety and depression in this specific population of medical staff in relation to the adverse stressors which were present during the COVID-19 pandemic. There is also evidence that the flourishing of both conditions altogether is not exclusive to medical staff, and there is generally a high probability of comorbidity in both disorders (35, 36). Furthermore, medical staff suffered from varying degrees of insomnia symptoms, including insomnia-early, insomnia-middle, and insomnia-late in agreement with the report of a higher percentage of medical staff experiencing sleep problems compared with other occupational groups during the past 3 months (37). Therefore, for the welfare and improving immunity of medical staff against the virus, work units should arrange reasonable working times, and ensure that such staff have adequate sleep quality.

We investigated the related factors of adverse psychological reactions in frontline and non-frontline medical staff, respectively. For frontline medical staff, we discovered that the marital status was connected with anxiety and the education level, history of contact with patients with COVID-19 and working in COVID-19 unit were connected with insomnia. These adverse reactions were more severe among frontline medical staff, possibly due to a skyrocketing workload, worrying about infected virus from COVID-19 patients and fearing of transmitting the virus to family numbers. For non-frontline medical staff, the age, and the marital status were not only related to anxiety, but also related to depression. However, the sex was related to anxiety and the education level was related to depression. In addition, the sex, the age, the marital status, the educational level, the history of contact with patients with COVID-19 and working in a COVID-19 unit were associated with the occurrence of insomnia. It was suggested that the marital status was a common associated factor for anxiety and the education level, history of contact with patients with COVID-19 and working in COVID-19 unit were common associated factors for insomnia in two medical groups. Besides, the adverse psychological reactions of non-frontline workers may be influenced by more factors, such as, the sex and age. Hence, we should pay attention to the mental health of all medical staff who have direct or indirect contact with the COVID-19 patients, giving targeted guidance on mental health.

We also explored the underlying factors that influence adverse psychological reactions. In particular, spinsterhood people were more prone to anxiety among frontline medical staff. What's more, having a bachelor's degree and a contact history of COVID-19 patient were more likely to suffer from insomnia among frontline medical staff. As expected, men were less susceptible to anxiety than women in non-frontline medical staff, which is consistent with a number of previous studies (15, 38–40). However, working at a COVID-19 unit were a risk factors for insomnia among the non-frontline group, but not among frontline group, which is inconsistent with the report of Su et al. (13) that SARS unit nurses had higher proportions of insomnia compared to non-SARS unit nurses during the SARS epidemic in Taiwan. Furthermore, we found that people in the middle age group were at lower risk for anxiety and depression in non-frontline medical staff, which was in line with previous research, which reported that older respondents were less susceptible to anxiety and depression disorder than younger people (13, 37). It was suggested that middle age group medical workers have more experience in epidemics than younger health workers. They are more psychologically resilient and may play a vital role in this epidemic.

Generally speaking, the sudden outbreak of COVID-19 led to increased workload, reduced rest time, worry about family infection, and reduced family activities for medical staff, which may have contributed to the presentation of mental health problems (41, 42). Previous studies have shown that at least 50% of medical staff needed psychological assistance (33, 43), which is consistent with the present study. Hence, it is vital for medical staff to obtain appropriate psychological aid and care. As we know, choosing the best way to conduct psychological counseling achieves the most satisfactory effects, and the development of internet technology is of great benefit and allows adoption of online forms for conducting psychological aid (41). By using this approach, we could not only effectively reduce the risk of virus transmission, but also increase crowd participation. In our survey, we concluded that medical staff preferred forms of WeChat or QQ group counseling, and public account publicity rather than face-to-face counseling. In addition, we sought to understand what psychological knowledge medical staff needed in order to provide targeted guidance. In our study, “how to self-alleviate psychological reactions,” “how to help others relieve psychological reactions” and “common psychological reactions” were the most popular contents for medical staff, and we should carry out more psychological knowledge guidance and training in these areas.

In this study, we had a sufficiently large sample size for a proper statistical analysis. The application of a PSM to eliminate the influence of confounding factors improved the authenticity and reliability of the conclusions, and the use of validated questionnaires and assessment of the value of psychological aids also contribute to the significance of the research. However, there were some limitations in the present study. First, this was a cross-sectional survey and our participants were not followed up. Thus, it is difficult to know how their mental health state will alter during the development of the COVID-19 pandemic, and a longitudinal study is needed to investigate the psychological effects on this population in future. Second, our data were collected *via* WeChat, DingTalk, and other social platforms, and the limitation of using social media for distributing questionnaires (i.e., medical staff who didn't use these social software were not enrolled in this study), may bias the results. Also, the clinical variables recollected in an online platform may not be entirely reliable, but this was the only way to collect the data because of confinement as a result of precaustions against the spread of COVID-19. Finally, the subjects enrolled in the present study were all medical staff and mostly from a low-risk zone. Previous studies have focused on participants in the high-risk zone, and there was no previous study sample from a low-risk zone. Nevertheless, the present study shows that the medical staff from a low-risk zone, especially frontline staff, experienced anxiety, depression, and insomnia as a result of the COVID-19 pandemic, and thus suggests that attention should be also paid to the mental health of medical staff in the low-risk zone.

Overall, the COVID-19 outbreak resulted in medical staff suffering from increases in certain mental health problems, and frontline medical staff were at greater risk for adverse psychological reactions than non-frontline staff. Identifying the underlying factors may contribute to the formulation of effective measures for relieving anxiety, depression, and insomnia symptoms among medical staff. Finally, we expect government and health systems to focus increasingly on the mental health of medical staff, especially the frontline group, and mental health care should have an indispensable role in global epidemic prevention and control.

## Data Availability Statement

The raw data supporting the conclusions of this article will be made available by the authors, without undue reservation.

## Ethics Statement

Informed consent was obtained from each participant, and the study was approved by the Ethics Committee of the Second Affiliated Hospital of Guangxi Medical University.

## Author Contributions

BG analyzed the data and contributed to the interpretation of results. WG, XM, and QS designed the study. QS, XM, and SGL completed the data collection. MY and WG made critical revisions and approved the final manuscript. All the authors including SL, QS, and XM wrote the first draft of the manuscript.

## Conflict of Interest

The authors declare that the research was conducted in the absence of any commercial or financial relationships that could be construed as a potential conflict of interest.
