# Supplementary material for: Adverse Psychological Reactions and Psychological Aids for Medical Staff During the COVID-19 Outbreak in China
Source: Front Psychiatry. 2021 Apr 15;12:580067. doi: 10.3389/fpsyt.2021.580067 (PMC8082095; doi:10.3389/fpsyt.2021.580067)
Supplement: Supplementary file 1 [file Table_1.doc]

| **TABLE S1** | Factors of influencing adverse psychological reactions in non-frontline group by logistic regression analysis | | | | | |  |
| --- | --- | --- | --- | --- | --- | --- |
| Anxiety/depression | β | SE | Wald | OR | OR95%CI | *P* |
| **Anxiety** |  |  |  |  |  |  |
| Sex |  |  |  |  |  |  |
| Men(control) |  |  |  |  |  |  |
| Women | 0.40 | 0.16 | 6.00 | 1.49 | 1.08-2.06 | **0.01** |
| Marital status |  |  |  |  |  |  |
| Widowed/divorced(control) |  |  |  |  |  |  |
| Married | 0.27 | 0.31 | 0.76 | 1.31 | 0.72-2.38 | 0.38 |
| Spinsterhood | 0.001 | 0.14 | 0.00 | 1.00 | 0.77-1.31 | 1.00 |
| Age |  |  |  |  |  |  |
| ≤28(control) |  |  |  |  |  |  |
| 29-40 | -0.35 | 0.17 | 4.09 | 0.70 | 0.50-0.99 | **0.04** |
| ＞40 | -0.05 | 0.13 | 0.15 | 0.95 | 0.73-1.24 | 0.70 |
| **Depression** |  |  |  |  |  |  |
| Age |  |  |  |  |  |  |
| ≤28(control) |  |  |  |  |  |  |
| 29-40 | -0.44 | 0.19 | 5.46 | 0.65 | 0.45-0.93 | **0.02** |
| ＞40 | -0.15 | 0.14 | 1.17 | 0.86 | 0.65-1.13 | 0.28 |
| Education level |  |  |  |  |  |  |
| <Undergraduate/junior college(control) |  |  |  |  |  |  |
| Undergraduate/junior college | -0.02 | 0.26 | 0.01 | 0.98 | 0.59-1.62 | 0.93 |
| ≥Postgraduate | -0.28 | 0.17 | 2.79 | 0.76 | 0.55-1.05 | 0.10 |
| Marital status |  |  |  |  |  |  |
| Widowed/divorced(control) |  |  |  |  |  |  |
| Married | 0.36 | 0.32 | 1.31 | 1.44 | 0.77-2.68 | 0.25 |
| Spinsterhood | 0.01 | 0.15 | 0.002 | 1.01 | 0.75-1.34 | 0.96 |
| **Insomnia-early** |  |  |  |  |  |  |
| Sex |  |  |  |  |  |  |
| Men(control) |  |  |  |  |  |  |
| Women | 0.38 | 0.13 | 8.70 | 1.47 | 1.14-1.89 | **0.003** |
| Marital status |  |  |  |  |  |  |
| Widowed/divorced(control) |  |  |  |  |  |  |
| Married | 0.23 | 0.27 | 0.72 | 1.26 | 0.74-2.14 | 0.40 |
| Spinsterhood | -0.23 | 0.09 | 6.50 | 0.80 | 0.67-0.95 | **0.01** |
| COVID-19 work unit |  |  |  |  |  |  |
| Negative(control) |  |  |  |  |  |  |
| Positive | 0.27 | 0.08 | 10.62 | 1.31 | 1.11-1.54 | **0.001** |
| **Insomnia-middle** |  |  |  |  |  |  |
| Sex |  |  |  |  |  |  |
| Men(control) |  |  |  |  |  |  |
| Women | 0.39 | 0.13 | 8.98 | 1.47 | 1.14-1.90 | **0.003** |
| COVID-19 work unit |  |  |  |  |  |  |
| Negative(control) |  |  |  |  |  |  |
| Positive | 0.26 | 0.08 | 9.87 | 1.30 | 1.10-1.52 | **0.002** |
| **Insomnia-late** |  |  |  |  |  |  |
| Age |  |  |  |  |  |  |
| ≤28(control) |  |  |  |  |  |  |
| 29-40 | -0.10 | 0.15 | 0.48 | 0.90 | 0.68-1.21 | 0.49 |
| ＞40 | -0.05 | 0.12 | 0.17 | 0.95 | 0.76-1.20 | 0.68 |
| Marital status |  |  |  |  |  |  |
| Widowed/divorced(control) |  |  |  |  |  |  |
| Married | 0.19 | 0.29 | 0.42 | 1.20 | 0.69-2.11 | 0.52 |
| Spinsterhood | -0.27 | 0.12 | 5.67 | 0.76 | 0.61-0.95 | **0.02** |
| Contact history |  |  |  |  |  |  |
| Negative(control) |  |  |  |  |  |  |
| Positive | -0.11 | 0.12 | 0.83 | 0.89 | 0.70-1.14 | 0.36 |
| COVID-19 work unit |  |  |  |  |  |  |
| Negative(control) |  |  |  |  |  |  |
| Positive | 0.31 | 0.09 | 13.41 | 1.37 | 1.16-1.61 | **<0.001** |
| **Sleep mode satisfaction** |  |  |  |  |  |  |
| Sex |  |  |  |  |  |  |
| Men(control) |  |  |  |  |  |  |
| Women | 0.46 | 0.16 | 8.61 | 1.58 | 1.17-2.15 | **0.003** |
| Education level |  |  |  |  |  |  |
| <Undergraduate/junior college(control) |  |  |  |  |  |  |
| Undergraduate/junior college | 0.44 | 0.30 | 2.23 | 1.55 | 0.87-2.77 | 0.14 |
| ≥Postgraduate | 0.24 | 0.18 | 1.80 | 1.27 | 0.90-1.79 | 0.18 |
| COVID-19 work unit |  |  |  |  |  |  |
| Negative(control) |  |  |  |  |  |  |
| Positive | 0.29 | 0.11 | 7.52 | 1.33 | 1.09-1.64 | **0.01** |
