# Supplementary material for: Adverse Psychological Reactions and Psychological Aids for Medical Staff During the COVID-19 Outbreak in China
Source: Front Psychiatry. 2021 Apr 15;12:580067. doi: 10.3389/fpsyt.2021.580067 (PMC8082095; doi:10.3389/fpsyt.2021.580067)
Supplement: Supplementary file 2 [file Data_Sheet_1.docx]

**A survey** **on mental health status and psychological aids for medical staff during the COVID-19 outbreak**

Dear medical staff:

Hello! This is a survey of mental health status and psychological aids for medical staff during the COVID-19 outbreak by the Mental Health Center of the Second Affiliated Hospital of Guangxi Medical University, China. This study was approved by the Ethics Committee of the Second Affiliated Hospital of Guangxi Medical University. The questionnaire is anonymous. You cannot be identified via the data analysis or manuscript when it is published. Please answer honestly and complete all the questions. Thank you very much!

The Mental Health Center of the Second Affiliated Hospital of Guangxi Medical University

1. Consent Statement: Did you volunteer to participate in this survey?

□Yes □No

2. How have you been feeling in the past two weeks?

2.1 I feel tense or 'wound up'

□Most of the time

□A lot of the time

□From time to time, occasionally

□Not at all

2.2 I still enjoy the things I used to enjoy:

□Definitely as much

□Not quite so much

□Only a little

□Hardly at all

2.3 I get a sort of frightened feeling as if something awful is about to happen:

□Very definitely and quite badly

□Yes, but not too badly

□A little, but it doesn't worry me

□Not at all

2.4 I can laugh and see the funny side of things:

□As much as I always could

□Not quite so much now

□Definitely not so much now

□Not at al

2.5 Worrying thoughts go through my mind:

□A great deal of the time

□A lot of the time

□From time to time, but not too often

□Only occasionally

2.6 I feel cheerful:

□Not at all

□Not often

□Sometimes

□Most of the time

2.7 I can sit at ease and feel relaxed:

□Definitely

□Usually

□Not often

□Not at all

2.8 I have lost interest in my appearance:

□Definitely

□I don't take as much care as I should

□I may not take quite as much care

□I take just as much care as ever

2.9 I feel restless as I have to be on the move:

□Very much indeed

□Quite a lot

□Not very much

□Not at all

2.10 I look forward with enjoyment to things:

□As much as I ever did

□Rather less than I used to

□Definitely less than I used to

□Hardly at all

2.11 I get sudden feelings of panic:

□Very often indeed

□Quite often

□Not very often

□Not at all

2.12 I feel as if I have slowed down:

□Nearly all the time

□Very often

□Sometimes

□Not at all

2.13 I get a sort of frightened feeling like 'butterflies' in the stomach:

□Not at all

□Occasionally

□Quite often

□Very often

2.14 I can enjoy a good book or radio or TV program:

□Often

□Sometimes

□Not often

□Very seldom

3. Please describe the severity of insomnia in the last week.

3.1 Insomnia-early

□ Never

□ Mild

□ Moderate

□ Severe

□ Extremely severe

3.2 Insomnia-middle

□ Never

□ Mild

□ Moderate

□ Severe

□ Extremely severe

3.3 Insomnia-late

□ Never

□ Mild

□ Moderate

□ Severe

□ Extremely severe

4. Were you satisfied with your sleep mode in the last month?

□ Always

□ Usually

□ A little

□ Seldom

□ Never

5. Do you think it is necessary to provide psychological aid?

□Yes □No

6. What forms of psychological aid do you prefer to receive?

□Hotline □WeChat or QQ group counseling

□Public account publicity  □Propaganda on TV and radio

□Received psychological information □Psychological counseling via video □Face-to-face psychological counseling □Group counseling with safeguards □Other

7. What contents of psychological aid do you want to receive?

□ Common psychological reactions

□ How to self-alleviate psychological reactions

□ How to help others relieve psychological reactions

□ Hhow to seek the help of a professional counselor

□ Not interested in

□ Other

8. Personal information

8.1 Your sex

□ Male □Female

8.2 Your age is

8.3 Your marital status

□ Widowed

□ Divorced

□ Married

□ Single

8.4 Your educational level

□ Junior high school and below

□ Senior high school/ secondary school

□ Undergraduate /junior college

□ Master

□ Doctor

8.5 You live in

8.6 You are

□ Frontline medical staff

□ Non-frontline medical staff

8.7 History of contact with patients with COVID-19

□ Positive

□ Negative

8.8 Work at the COVID-19 unit

□ Positive

□ Negative
