# Supplementary material for: Adverse Psychological Reactions and Psychological Aids for Medical Staff During the COVID-19 Outbreak in China
Source: Front Psychiatry. 2021 Apr 15;12:580067. doi: 10.3389/fpsyt.2021.580067 (PMC8082095; doi:10.3389/fpsyt.2021.580067)
Supplement: Supplementary file 3 [file Data_Sheet_2.docx]

新冠感染期间医务工作者心理状况与心理援助需求调查

尊敬的医务工作者:

您好！新冠感染疫情期间您辛苦了。这是我们对疫情下医务工作者的心理状况及心理援助需求的调查，数据只用于分析，个人数据和信息保密，答案无对错之分。恳请您如实作答并完成全部问题，为制定疫情期间的心理援助方案提供理论依据。希望得到您的支持和配合。不胜感激！本研究经广西医科大学第二附属医院伦理委员会批准。

广西医科大学第二附属医院心理卫生科

1. 您是否自愿参加此项调查？

□是 □否

2. 新冠感染疫情出现后，最近两周您的心情如何

2.1 我感到紧张或痛苦。

　□根本没有——0分

　□有时候——1分

　□大多时候——2分

　□几乎所有时候——3分

2.2我对以往感兴趣的事情还是有兴趣。

　□肯定一样——0分

　□不像以前那样多——1分

　□只有一点——2分

　□基本上没有了——3分

2.3我感到有点害怕，好像预感到什么可怕的事情要发生。

　□根本没有——0分

　□有一点，但并不使我苦恼——1分

　□是有，但不太严重——2分

　□非常肯定和十分严重——3分

2.4我能够哈哈大笑，并看到事物好的一面：

　□我经常这样——0分

　□现在已经不大这样了——1分

　□现在肯定是不大多了——2分

　□根本没有——3分

2.5我的心中充满烦恼。

　□偶然如此——0分

　□时时，但不经常——1分

　□常常如此——2分

　□大多数时间——3分

2.6我感到愉快。

　□大多数时间——0分

　□有时——1分

　□并不经常 ——2分

　□根本没有——3分

2.7我能够安静而轻松地坐着。

　□肯定——0分

　□经常——1分

　□并不经常——2分

　□根本没有——3分

2.8我对自己的仪容（打扮自己）失去兴趣：

　□我仍然像以往一样关心——0分

　□我可能不是非常关心——1分

　□并不像我应该做的那样关心——2分

　□肯定——3分

2.9我有点坐立不安，好像感到非要活动不可。

　□根本没有——0分

　□并不很多——1分

　□是不少——2分

　□确实非常多——3分

2.10我对一切都是乐观地向前看。

　□差不多是这样的——0分

　□并不完全是这样的——1分

　□很少这样——2分

　□几乎从不这样做——3分

2.11我突然发现有恐慌感：

　□根本没有——0分

　□并非经常——1分

　□时常 ——2分

　□确实很经常——3分

2.12我好像感到情绪在渐渐低落：

　□根本没有——0分

　□有时——1分

　□经常——2分

　□几乎所有时间——3分

2.13我感到有点害怕，好像某个内脏器官变坏了：

　□根本没有——0分

　□有时——1分

　□经常 ——2分

　□大多数时间——3分

2.14我能欣赏一本好书或一项好的广播或电视节目：

　□常常如此——0分

　□有时——1分

　□并非经常——2分

　□很少——3分

3描述您当前（或最近一周）失眠问题的严重程度:

3.1入睡困难

□无 □轻度 □中度 □重度 □极重度

3.2维持睡眠困难

□无 □轻度 □中度 □重度 □极重度

3.3早醒

□无 □轻度 □中度 □重度 □极重度

4.对您当前睡眠模式的满意度

□很满意 　□满意 　□一般 　□不满意 　□很不满意

5.您觉得心理援助有必要吗

□有 □无

6.您希望心理援助以什么方式开展（多选）

□热线电话 □微信或QQ群辅导 □公众号宣传

□电视、广播的宣传 □收到关于心理方面的资料 □视频心理辅导  □面对面心理辅导 □做好防护的团体心理辅导 □其它_________

7.您希望了解哪些有关心理方面知识和技能（多选）

□常见心理反应 □自己如何缓解心理反应 □如何帮助别人缓解心理反应 □如何寻求专业心理咨询人员的帮助 □不感兴趣 □其它_________

8.基本信息

8.1您的性别

□男 □女

8.2您的年龄___________岁

8.3婚姻状况

□未婚 □已婚 □丧偶 □离异

8.4学历

□初中及以下 □高中/中专 □本科/大专 □硕士 □博士

8.5您目前住在__________（选项）

8.6您是

□疫情一线医护人员 □非一线医务人员

8.7您接触过新型冠状病毒感染和或疑似患者吗？

□有 □无

8.8您工作单位有新型冠状病毒感染和或疑似患者吗？

□有 □无
